# Bmi1 regulate tooth and mandible development by inhibiting p16 signal pathway

**DOI:** 10.1111/jcmm.16468

**Published:** 2021-03-21

**Authors:** Ying Yin, Nan Zhou, Hui Zhang, Xiuliang Dai, Xianhui Lv, Ning Chen, Dengshun Miao, Qingang Hu

**Affiliations:** ^1^ Nanjing Stomatological Hospital Medical School of Nanjing University Nanjing China; ^2^ Department of Anatomy, Histology and Embryology State Key Laboratory of Reproductive Medicine The Research Center for Bone and Stem Cells Nanjing Medical University Nanjing China; ^3^ Department of Non‐communicable Disease Prevention Nanjing Municipal Center for Disease Control and Prevention Nanjing China; ^4^ Reproductive Center Nanjing Medical University Affiliated Changzhou Maternal and Child Health Care Hospital Changzhou China; ^5^ Institute of Stomatology Nanjing Medical University Nanjing China; ^6^ The Research Center for Aging Affiliated Friendship Plastic Surgery Hospital of Nanjing Medical University Nanjing China

**Keywords:** Bmi1, development, mandible, p16, tooth

## Abstract

To determine whether the deletion of p16 can correct tooth and mandible growth retardation caused by Bmi1 deficiency, we compared the tooth and mandible phenotypes of homozygous p16‐deficient (p16^−/−^) mice, homozygous Bmi1‐deficient (Bmi1^−/−^) mice, double homozygous Bmi1 and p16‐deficient (Bmi1^−/−^p16^−/−^) mice to those of their wild‐type littermates at 4 weeks of age by radiograph, histochemistry and immunohistochemistry. Results showed that compared to Bmi1^−/−^ mice, the dental mineral density, dental volume and dentin sialoprotein immunopositive areas were increased, whereas the ratio of the predentin area to total dentin area and that of biglycan immunopositive area to dentin area were decreased in Bmi1^−/−^p16^−/−^ mice. These results indicate that the deletion of p16 can improve tooth development in Bmi1 knockout mice. Compared to Bmi1^−/−^ mice, the mandible mineral density, cortical thickness, alveolar bone volume, osteoblast number and activity, alkaline phosphatase positive area were all increased significantly in Bmi1^−/−^p16^−/−^ mice. These results indicate that the deletion of p16 can improve mandible growth in Bmi1 knockout mice. Furthermore, the protein expression levels of cyclin D, CDK4 and p53 were increased significantly in p16^−/−^ mice compared with those from wild‐type mice; the protein expression levels of cyclin D and CDK4 were decreased significantly, whereas those of p27 and p53 were increased significantly in Bmi1^−/−^ mice; these parameters were partly rescued in Bmi1^−/−^p16^−/−^ mice compared with those from Bmi1^−/−^ mice. Therefore, our results indicate that Bmi1 plays roles in regulating tooth and mandible development by inhibiting p16 signal pathway which initiated entry into cell cycle.

## 1 INTRODUCTION

Bmi1 is a member of the family of polycomb (PcG) proteins. The PcG proteins are a group of transcriptional inhibitors that regulate the target gene by chromatin modification, which are closely related to the determinants of stem cell fate, X chromosome inactivation, cell differentiation and cell carcinogenesis. PcG proteins can be divided into two core protein complexes: the first is the multi‐comb inhibitory complex 2 (PRC2), which plays a role in the initial stage of transcription inhibition; the second is the multi‐comb inhibitory complex 1 (PRC1), which maintains the stability of inhibitory state chromatin.^1^, ^2^ Bmi1 is one of the PRC1 constituents and plays an important regulatory role in the self‐renewal of stem cells. Hosen et al^3^ reported that Bmi1 was highly expressed in hematopoietic stem cells and was downregulated upon commitment to differentiation. Bmi1 plays an important role in the proliferation of normal stem cells and precursor cells in the liver of the foetus.^4^ In addition, Bmi1 plays an important role in activating telomerase activity, thus preventing cell ageing and maintaining cell immortalization.^5^ In order to realize the in vivo function of Bmi1 gene, Nathalie et al established Bmi1 knockout (Bmi1^−/−^) mouse model by homologous recombination technique. The Bmi1^−/−^ mice displayed defects in haematopoiesis and development of the central and peripheral nervous systems. Bmi1^−/−^ mice were normal in size and appearance at birth; however, they exhibited progressive post‐natal growth retardation and died by early adulthood with signs of hematopoietic failure and neurological abnormalities.^6^, ^7^, ^9^ It was reported that Bmi1^−/−^ mice had abnormal axial bone morphogenesis and increased bone marrow adipocytes.^8^, ^10^

Bmi1 maintains cell self‐renewal and cell cycle progression to prevent cell senescence by inhibiting the transcription of the ink4a‐arf gene encoding the cell cycle‐dependent kinase inhibitory factor (CDKI).^11^ A recent study has demonstrated that Bmi1 can bind directly to the Bmi1‐responding element of the p16 promoter to repress its expression.^12^ The ink4a‐arf gene overlaps two antitumor proteins: p16^ink4a^ (p16) and p19^arf^ (p19). p16 selectively inhibits cyclin‐dependent kinase 4/6 (CDK4/CDK6), thereby inhibiting phosphorylation of retinoblastoma protein (Rb). Rb inhibits downstream gene expression required for translation from the G1 phase into the S phase by binding to the cell cycle‐related gene transcription factor E2F, resulting in cell growth arrest.^13^, ^14^ Studies showed that p16‐positive cell deletion could significantly increase the weight of mice, muscle fibre diameter and fat layer thickness, thus delaying the ageing process.^15^ As one of the downstream targets of Bmi1, knockout of p16 gene partially corrected the ability of neural stem cells and hematopoietic stem cells to self‐renew.^13^, ^16^, ^17^

It was reported that knockout of p16 and p19 genes in Bmi1^−/−^ mice improved the number, proliferation and activity of stem cells in Bmi1‐deficient incisors, indicating that knockout of p16 and p19 partially rescued Bmi1‐deficient incisor stem cell dysfunction.^18^ We previously reported that Bmi1 deficiency resulted in defects in dentin and alveolar bone formation, and p16 was increased in Bmi1‐deficient mandible.^19^, ^20^ However, the role of p16 in dentin development and mandibular osteogenesis in Bmi1^−/−^ mouse was unclear.

In this study, we showed that Bmi1 gene expression was downregulated with age, while p16 gene only expressed in the old stage. To investigate whether the function of Bmi1 was mediated through p16 in regulating tooth and mandible development, compound mutant mice with homozygous deletion of both Bmi1 and p16 (Bmi1^−/−^p16^−/−^) were generated. Their mandible phenotype was then compared with p16^−/−^, Bmi1^−/−^ and wild‐type mice at 4 weeks of age.

## 2 MATERIALS AND METHODS

### 2.1 Mice and genotyping

p16^Ink4a+/−^ mice of the FVB N2 background were crossed to Bmi1^+/−^ (129Ola/FVB/N hybrid background) mice to generate WT, Bmi1^−/−^, p16^−/−^ and double‐knockout Bmi1^−/−^p16^−/−^ mice and genotyped as described previously.^21^ Wild‐type Bmi1 and p16 allele were amplified with Bmi1 forward primer (5′‐CAGTTAGGCAGTATGTAGTTTTC‐3′) and p16 forward primer (5′‐GGCAAATAGCGCCACCTAT‐3′); Bmi1 reverse primer (5′‐GTTGTGGTGGAGTGTAAGAGTGT‐3′) and p16 reverse primer (5′‐GACTCCATGCTGCTCCAGAT‐3′). To detect the presence of the null Bmi1 and p16 allele, the neomycin gene was detected with the primers neo‐F (5′‐AAGATGTTGGCGACCTCGTATTGG‐3′) and neo‐R (5′‐GCAAGACCTGCCTGAAACCGAACT‐3′) for Bmi1, while neo‐F (5′‐GCCGCTGGACCTAATAACTTC‐3′) and neo‐R (5′‐GACTCCATGCTGCTCCAGAT‐3′) for p16. Four‐week‐old WT, Bmi1^−/−^, p16^−/−^ and Bmi1^−/−^ p16^−/−^ mice used in the present study. Each group contained five animals. This study was carried out in accordance with the guidelines of the Institute for Laboratory Animal Research of Nanjing Medical University in Nanjing of China. The protocol was approved by the Committee on the Ethics of Animal Experiments of Nanjing Medical University (Permit Number: IACUC‐1706001).

### 2.2 Radiography

Mandibles were fixed in PLP fixative (2% paraformaldehyde containing 0.075 mol/L lysine and 0.01 mol/L sodium periodate) overnight at 4°C. A Faxitron model 805 radiographic inspection system (Faxitron, München, Germany) took using contact radiographs, at 22 kV voltage and with a 4‐minute exposure time. X‐Omat TL film (Eastman Kodak, Rochester, NY, USA) was used and processed routinely.

### 2.3 Micro‐computed tomography (micro‐CT)

Mandibles were fixed in PLP fixative (2% paraformaldehyde containing 0.075 mol/L lysine and 0.01 mol/L sodium periodate) overnight at 4°C. Then the samples were analysed by micro‐CT with a SkyScan 1072 scanner and associated analysis software (SkyScan, Antwerp, Belgium) as described.^19^ Briefly, the samples were enclosed in tightly fitting plastic wrap to prevent movement and dehydration. Thresholding was applied to the images to segment the bone from the background. Two‐dimensional images were used to generate three‐dimensional renderings using the 3D Creator software supplied with the instrument. The resolution of the micro‐CT images is 18.2 μm.

### 2.4 Histology

Mandibles were fixed in PLP fixative overnight at 4°C and processed histologically as described.^19^ Mandibles were decalcified in EDTA‐glycerol solution for 7‐10 days at 4°C. Decalcified right mandibles were dehydrated and embedded in paraffin, and 5 μm sections were cut on a rotary microtome. The sections were stained with haematoxylin and eosin (HE), or histochemically for total collagen and ALP, or immunohistochemically as described below.

### 2.5 Immunohistochemical staining

Immunohistochemical staining was carried out for biglycan and dentin sialoprotein (DSP), using the avidin‐biotin‐peroxidase complex technique with affinity‐purified rabbit anti‐mouse biglycan antibody (1:400, ab58562, Abcam, Cambridge, UK) and dentin sialoprotein (1:200, sc‐33587, Santa Cruz, CA, USA) following previously described methods.^19^ Briefly, deparaffinized and rehydrated sections were brought to a biol in 10 mmol/L sodium citrate buffer pH6.0 for antigen retrieval. And then the sections were incubated with 3% hydrogen peroxide to block endogenous peroxidase activity and then washed in Tris‐buffered saline (pH 7.6). The slides were incubated with the primary antibodies overnight at 4°C. After rinsing with Tris‐buffered saline for 5 minutes for 3 times, slides were incubated with secondary antibody. Then Vectastain Elite ABC reagent (Vector Laboratories, Burlingame, CA, USA) was used to incubated for 45 minutes. Staining was developed using 3, 3‐diaminobenzidine (2.5 mg/mL) followed by counterstaining with Mayer's haematoxylin.

### 2.6 Western blot analysis

Proteins were extracted from mandibles and quantitated using a protein assay kit (Bio‐Rad, Mississauga, Ontario, Canada). Protein samples (20 μg) were fractionated by SDS‐PAGE and transferred to nitrocellulose membranes. Immunoblotting was carried out as described^19^ using antibodies against Cyclin D (1:300, sc‐8396, Santa Cruz), CDK4 (1:300, sc‐23896, Santa Cruz), p27 (1:500, sc‐1641, Santa Cruz), p53 (1:500, sc‐126, Santa Cruz) and β‐actin (1:1000, AP0714, Bioworld Technology). Bands were visualized using ECL chemiluminescence (Amersham) and quantitated by Scion Image Beta 4.02 (Scion Corporation, NIH).

### 2.7 Computer‐assisted image analysis

After HE staining or histochemical or immunohistochemical staining of sections from five mice of each genotype, images of fields were photographed with a Sony digital camera. Images of micrographs from single sections were digitally recorded using a rectangular template, and recordings were processed and analysed using Northern Eclipse image analysis software as described.^19^

### 2.8 Statistical analysis

Data from image analysis are presented as mean ± SEM. Statistical comparisons were made using a two‐way ANOVA, with *P* < .05 considered significant.

## 3 RESULTS

### 3.1 p16 knockout improved teeth and mandible growth and mineralization of Bmi1‐deficient mice

**FIGURE 1:**
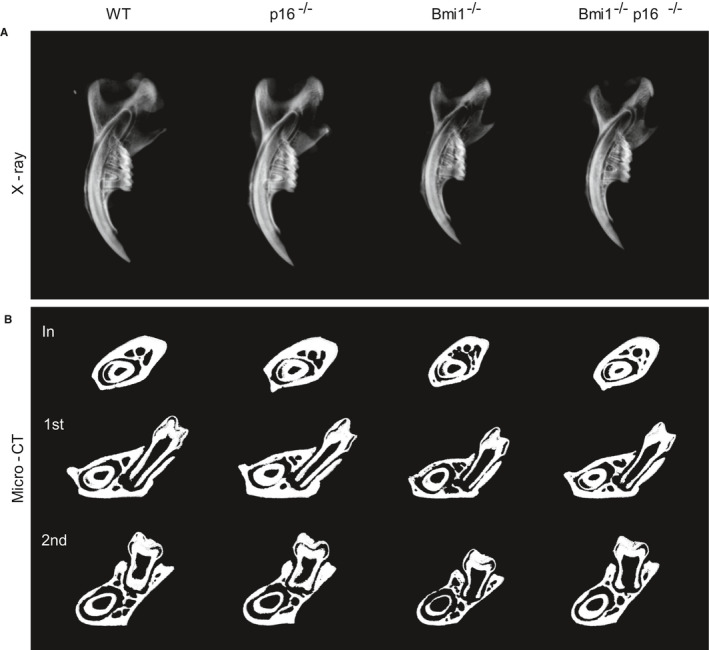
p16 knockout improved teeth and mandible growth and mineralization of Bmi1‐deficient mice. (A) Representative contact radiographs of the mandibles from 4‐wk‐old wild‐type (WT), p16^−/−^, Bmi1^−/−^ and Bmi1^−/−^p16^−/−^ mice. (B) Micro‐CT scanned sections through the incisor before the first molar (In), and through the first (1st) and second (2nd) molars from WT, p16^−/−^, Bmi1^−/−^ and Bmi1^−/−^p16^−/−^ mice

To clarify the effect of p16 knockout on the growth and mineralization of Bmi1^−/−^ mice, differences in mandible general morphology and bone mineral density among 4‐week‐old WT, p16^−/−^, Bmi1^−/−^ and Bmi1^−/−^p16^−/−^ mice were compared by X‐ray and micro computed tomography (micro‐CT). Bone mineral density was increased in p16^−/−^ mice compared to the 4‐week‐old WT littermates, while it was decreased in the Bmi1^−/−^ and Bmi1^−/−^p16^−/−^ mice. Compared with Bmi1^−/−^ mice, bone mineral density of Bmi1^−/−^p16^−/−^ mice was significantly increased (Figure 1A).

Mandible specimens of the 4‐week‐old mice were compared by micro‐CT through the incisor before the first molar and the first and second molars. The results showed: the cortical bone and trabecular bone volume, the volume of the incisors, the first and the second molars and the thickness of the root canal wall were slightly increased in p16^−/−^ mice compared to the 4‐week‐old WT littermates, while they were all decreased in the Bmi1^−/−^ and Bmi1^−/−^p16^−/−^ mice; while compared with the Bmi1^−/−^ mice, the cortical bone and trabecular bone volume, the volume of the incisors, the first and the second molars and the thickness of the root canal wall were all increased in the Bmi1^−/−^p16^−/−^ mice (Figure 1B).

These results suggested that p16 gene knockout can partially correct the growth and mineralization of dental and mandible of Bmi1^−/−^ mice.

### 3.2 p16 knockout increased the tooth volume and alveolar bone volume in Bmi1‐deficient mice

**FIGURE 2:**
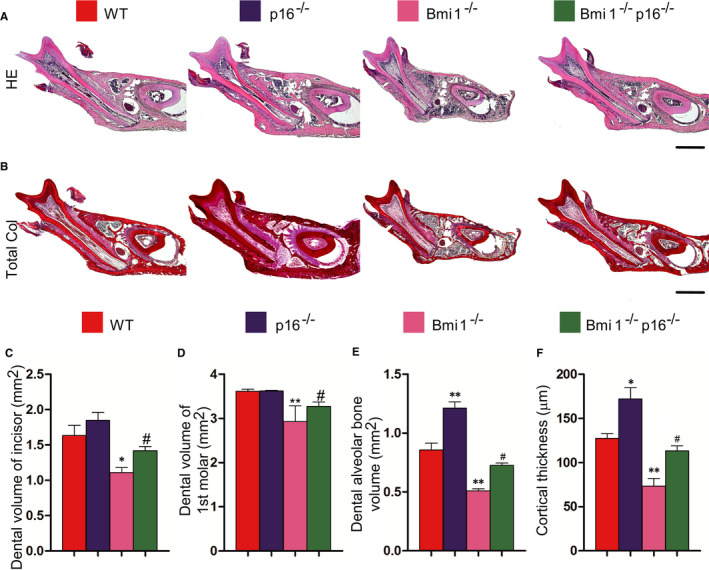
p16 knockout increased the tooth volume and alveolar bone volume in Bmi1‐deficient mice. Paraffin‐embedded sections through the first molars from 4‐wk‐old wild‐type (WT), p16^−/−^, Bmi1^−/−^ and Bmi1^−/−^p16^−/−^ mice stained with (A) HE, and with (B) serious red for total collagen. Scale bars represent 400 μm. (C) Dental volume of incisors (mm^2^), (D) dental volume of the first molars (mm^2^), (E) dental alveolar bone volume (mm^2^) and (F) cortical thickness (μm) were measured. Each value is the mean ± SEM of determinations in five animals of each group. *P < .05 relative to the wild‐type mice

In order to clarify the effect of p16 knockout on the tooth volume and alveolar bone volume in Bmi1^−/−^ mice, HE and total collagen histochemical staining was performed on the paraffin section of the first molars. HE and total collagen histochemical staining analysis showed that compared with 4‐week‐old WT mice, the dental volume of the incisors and first molars, alveolar bone volume and cortical bone thickness were reduced in Bmi1^−/−^ and Bmi1^−/−^p16^−/−^ mice. These parameters in Bmi1^−/−^p16^−/−^ mice were significantly increased compared with those in the Bmi1^−/−^ mice (Figure 2). These results suggested that p16 gene deletion partially corrected the developmental defect of tooth volume and alveolar bone volume in Bmi1‐deficient mice.

### 3.3 p16 knockout promoted dentin maturation and formation in Bmi1‐deficient mice

**FIGURE 3:**
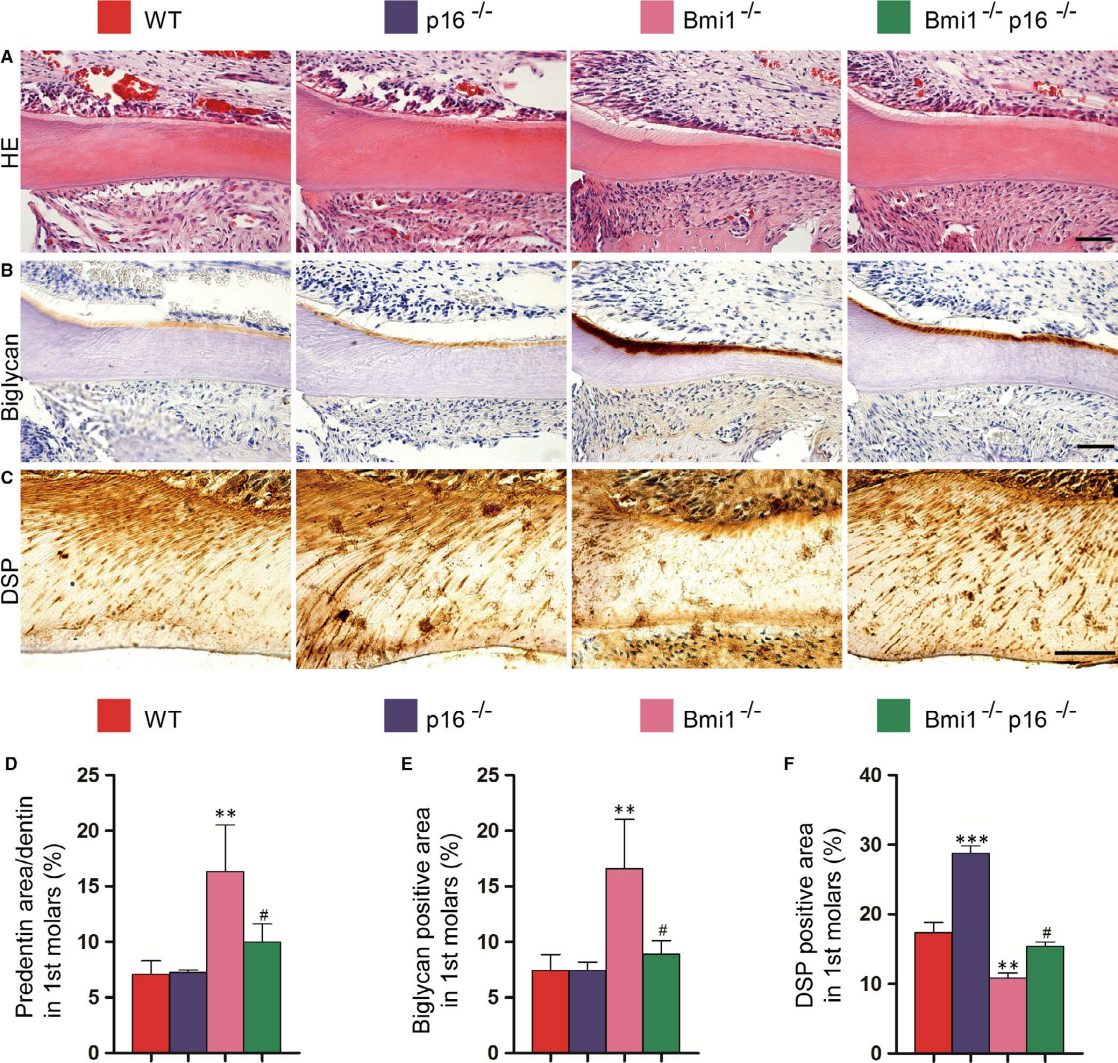
p16 knockout promoted dentin maturation and formation in Bmi1‐deficient mice. Paraffin‐embedded sections through the first molars and the incisors from 4‐wk‐old wild‐type (WT), p16^−/−^, Bmi1^−/−^ and Bmi1^−/−^p16^−/−^ mice stained with (A) HE, immunohistochemically for (B) biglycan and (C) dentin sialoprotein (DSP) and photographed. Scale bars represent 50 μm. (A) Representative HE staining of micrographs of the root walls of the first molars. (D) Quantitative thickness of predentin in the root walls of the first molars. (B) Representative micrographs of the root wall of the first molars stained immunohistochemically for biglycan, and (C) the root wall of the first molars stained immunohistochemically for DSP. (E) Quantitative biglycan immunopositive areas in the first molars. (F) Quantitative DSP immunopositive areas in the first molars. Each value is the mean ± SEM of determinations in five animals of each group. **P < .01; ***P < .001 relative to the wild‐type mice. #*P* < .05 compared with Bmi1^−/−^ mice

In order to clarify the effect of p16 knockout on dentin maturation and formation in Bmi1^−/−^ mice, HE staining was performed on the paraffin section of the first molar. The ratio of predentin to total dentin was significantly increased in the Bmi1^−/−^ mice compared with the WT mice, while it was significantly decreased in the Bmi1^−/−^p16^−/−^ mice compared with that of the Bmi1^−/−^ mice (Figure 3A,D). Biglycan positive area was increased in the Bmi1^−/−^ and Bmi1^−/−^p16^−/−^ mice compared with that of WT littermates, whereas it was significantly reduced in the Bmi1^−/−^p16^−/−^ mice compared with that of Bmi1^−/−^ mice (Figure 3B,E). DSP positive area ratio in the first molar in Bmi1^−/−^ and Bmi1^−/−^p16^−/−^ mice was significantly decreased compared with that of the 4‐week‐old WT littermates, whereas it was significantly increased in the Bmi1^−/−^p16^−/−^ mice compared with that of Bmi1^−/−^ mice (Figure 3C,F). These results indicated that the deletion of p16 gene partially corrected the dentin maturation and formation disorder in Bmi1 gene‐deficient mice.

### 3.4 p16 knockout accelerated formation of alveolar bone in Bmi1‐deficient mice

**FIGURE 4:**
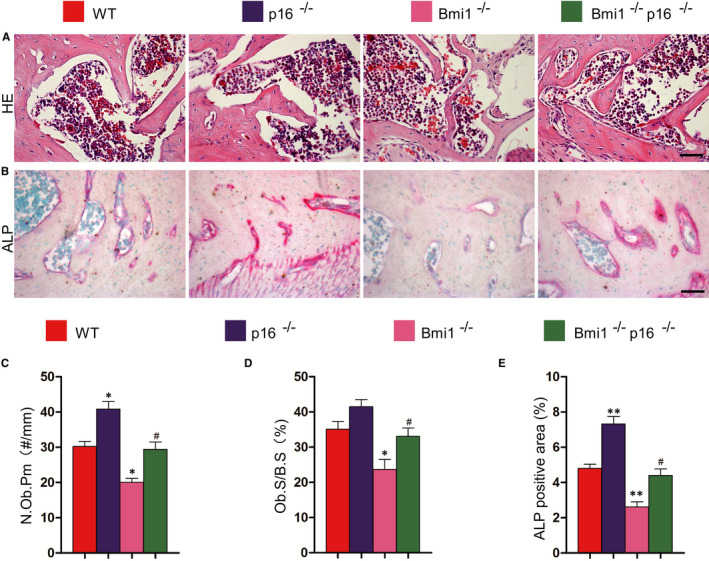
p16 knockout accelerated formation of alveolar bone in Bmi1‐deficient mice. Paraffin‐embedded sections through the first molars and the incisors from 4‐wk‐old wild‐type (WT), p16^−/−^, Bmi1^−/−^ and Bmi1^−/−^p16^−/−^ mice stained with (A) HE and histochemically for (B) ALP. Scale bars represent 50 μm. (C) Number of osteoblasts per mm bone parameter (N. Ob/B. Pm, #/mm) and (D) the surface of osteoblasts relative to the bone surface (Ob.S/B.S, %) were determined in the dental alveolar bone of HE‐stained mandibles, respectively. (E) ALP positive area as a percentage of the tissue area in the dental alveolar bone. *P < .05; **P < .01 relative to the wild‐type mice. #*P* < .05 compared with Bmi1^−/−^ mice

In order to clarify whether the effect of p16 gene knockout on osteoblastic alveolar bone formation in Bmi1^−/−^ mice, HE and ALP histochemical staining was performed on the paraffin section. The results showed that alveolar osteoblast cells and osteoblast surface were significantly decreased in the Bmi1^−/−^ and Bmi1^−/−^p16^−/−^ mice compared with those of WT mice, whereas they were significantly increased in the Bmi1^−/−^ p16^−/−^ mice compared with those of the Bmi1^−/−^ mice (Figure 4A,C,D). ALP positive area of trabecular bone in alveolar bone in the Bmi1^−/−^ mice was decreased in the Bmi1^−/−^ and Bmi1^−/−^p16^−/−^ mice compared with that of WT mice; however, ALP positive area in the Bmi1^−/−^p16^−/−^ mice was significantly increased compared with that of the Bmi1^−/−^ mice (Figure 4B,E). These results indicated that p16 gene deletion partially corrected the osteoblastic bone formation caused by deletion of Bmi1 gene, thus promoting the increase in alveolar bone volume.

### 3.5 p16 knockout regulated cell cycle‐related factor protein expression in Bmi1‐deficient mandible

**FIGURE 5:**
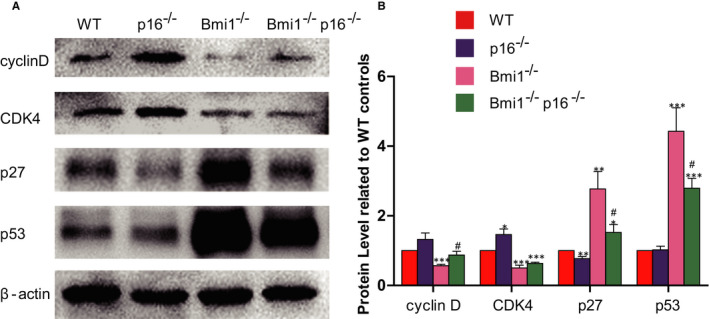
p16 knockout regulated cell cycle‐related factor protein expression in Bmi1‐deficient mandible. (A) Western blots of mandible extracts were carried out for expression of cyclin D, CDK4, p27 and p53, with β‐actin as a loading control. (B) Histograms showing normalized protein quantification for cyclin D, CDK4, p27 and p53. *P < .05; **P < .01; ***P < .001 relative to the wild‐type mice. #*P* < .05 compared with Bmi1^−/−^ mice

To clarify whether p16 gene knockout was associated with changes in cell cycle‐related factors, the expression of cell cycle‐related factors of dental and mandible proteins were analysed by Western blot. Protein expressions of the Cyclin D and CDK4 in the Bmi1^−/−^ mice were significantly downregulated, while p27 and p53 were upregulated compared with that of WT mice; these four indicators in the Bmi1^−/−^p16^−/−^ mice were rescued in a certain degree; however, expressions of them did not reach the level of WT mice (Figure 5).

## 4 DISCUSSION

In this study, p16 was a cell cycle‐dependent kinase inhibitor, knockout of p16 gene could partially correct the abnormal expression of cell cycle‐related factors caused by Bmi1 deletion, thereby promoting the cell cycle process and playing a role in correcting teeth and mandibular dysplasia caused by Bmi1 deletion.

p16 is an important downstream target of Bmi1, which is significantly upregulated in Bmi1‐deficient teeth and Bmi1‐deficient mandible.^12^, ^19^, ^20^ Bmi1 may regulate the development of teeth and mandible by inhibiting the expression of p16. In order to clarify the role and mechanism of p16 on teeth and mandible in Bmi1‐deficient mice, phenotypes of the teeth and mandible in 4‐week‐old WT mice, p16^−/−^ mice, Bmi1^−/−^ mice, Bmi1^−/−^p16^−/−^ mice were examined.

We first analysed the effect of p16 on tooth developmental disorder in Bmi1^−/−^ mice by imaging and histochemical staining. Results showed that knockout of p16 gene could increase the mandible volume, the degree of mineralization, the incisor and the first molar volume in Bmi1^−/−^ mice. Dentin is the main component of tooth. Analysis of dentin formation revealed that knockout of the p16 gene reduced the ratio of predentin and the ratio of Biglycan‐positive area, but it increased the positive area of DSP in Bmi1‐deficient mice. These results suggested that knockout of p16 gene was able to improve the dentin mineralization maturation disorder due to Bmi1 deletion. Previous studies have shown that the number of Bmi1‐deficient apical stem cells was reduced, the ability of the lingual cervical ring cells to enamel differentiation was weakened, and the deposition of enamel was reduced. Ink4a/Arf deletion can restore the capacity and the number of lumbar neck rings, but was unable to improve enamel developmental barrier.^18^ It has been reported that the expression level of p16 gene was increased by 5 to 21 times in the central nervous system and peripheral nervous system of Bmi1 deficiency in all ages, and the gene expression level of p19 was only increased by 1.4‐3 times.^22^ Therefore, we believed that p16 played an important role in the nervous system development and maintenance with Bmi1 deletion. Teeth are derived from the differentiation of neural crest cells. p16 deletion can significantly improve by dentin development disorders caused by Bmi1 deficiency, which may be related to the ability that p16 deletion can restore the expression of DSP. DSP is a specific protein produced by odontoblast cells during odontoblast differentiation.^23^ The expression of DSP in Bmi1^−/−^ teeth was significantly reduced, indicating that the function of odontoblast cells was impaired. The recovery of DSP expression after p16 knockout suggested that the function of odontoblast cells was corrected.

Previous studies have shown that the deficiency of Ink4a/Arf failed to correct the phenotype of osteoblast formation and proliferation caused by deletion of Bmi1.^16^ However, the lack of Ink4a/Arf was able to partially correct the neurological developmental disorder resulted from Bmi1 deletion.^13^, ^17^ We doubted whether p16 gene knockout can improve the neural crest cells developed from the mandible phenotype in Bmi1‐deficient mice. Our results showed that knockout p16 can increase the ratio of alveolar bone, osteoblast number, osteoblast surface and ALP activity in Bmi1^−/−^ mice, and suggested that p16 gene knockout can increase the ability to bone formation in Bmi1‐deficient osteoblasts.

p16 is one of the cell cycle‐dependent kinase inhibitory factors and plays an important role in the regulation of cell cycle. p16 can selectively inhibit cyclin‐dependent kinase 4/6 (CDK4/CDK6), thus inhibiting the phosphorylation of retinoblastoma protein (Rb). Rb in the non‐phosphorylation of the state shields the transcriptional activation domain by binding the cell cycle‐related gene transcription factor E2F, thus inhibiting downstream gene expression from the G1 phase to the S phase, resulting in cell growth stagnation.^14^ p27 as one of cyclin‐dependent kinase inhibitors is a negative regulatory factor to mandible formation.^24^ The activation of p53 leads to the regulation of cell fate outcomes, such as growth arrest and apoptosis, by controlling the expression of target genes.^25^ Detection of cell cycle‐related factors found that protein expressions of the Cyclin D and CDK4 in the Bmi1^−/−^ mice were significantly downregulated, while p27 and p53 were up‐regulated. These four indicators in the Bmi1^−/−^p16^−/−^ mice were rescued in a certain degree; however, expressions of them were still not as the same as in the WT mice. The above results showed that knockout of p16 gene can partially correct Bmi1 deletion caused by the abnormal expression of cell cycle‐related factors, thereby promoting the cell cycle process and playing a role in correcting teeth and mandible dysplasia caused by Bmi1 deletion.

In summary, results of this study showed that Bmi1 can inhibit p16 and promote cell cycle progression, thus playing a role in promoting the growth of teeth and mandible.

## CONFLICT OF INTEREST

The authors confirm that there are no conflicts of interest.

## AUTHOR CONTRIBUTIONS

**Ying Yin:** Conceptualization (equal); data curation (lead); funding acquisition (equal); project administration (equal); writing‐original draft (lead); writing‐review & editing (lead). **Nan Zhou:** Formal analysis (lead); methodology (supporting); resources (equal). **Hui Zhang:** Data curation (equal); methodology (supporting); software (supporting). **Xiuliang Dai:** Resources (supporting); writing‐review & editing (supporting). **Xianhui Lv:** Data curation (supporting). **Ning Chen:** Software (equal); supervision (equal). **Dengshun Miao:** Conceptualization (lead); funding acquisition (equal); project administration (lead); supervision (equal); writing‐review & editing (lead). **Qingang Hu:** Project administration (lead); supervision (equal); writing‐original draft (lead); writing‐review & editing (equal).

## Data Availability

The data that support the findings of this study are available from the corresponding author upon reasonable request.

## References

[1] Lund AH , van Lohuizen M . Polycomb complexes and silencing mechanisms. Curr Opin Cell Biol. 2004;16(3):239‐246. 15145347 10.1016/j.ceb.2004.03.010

[2] Otte AP , Kwaks TH . Gene repression by Polycomb group protein complexes: a distinct complex for every occasion? Curr Opin Genet Dev. 2003;13(5):448‐454. 14550408 10.1016/s0959-437x(03)00108-4

[3] Hosen N , Yamane T , Muijtjens M , Pham K , Clarke MF , Weissman IL . Bmi‐1‐green fluorescent protein‐knock‐in mice reveal the dynamic regulation of bmi‐1 expression in normal and leukemic hematopoietic cells. Stem Cells. 2007;25(7):1635‐1644. 17395774 10.1634/stemcells.2006-0229

[4] Lessard J , Sauvageau G . Bmi‐1 determines the proliferative capacity of normal and leukaemic stem cells. Nature. 2003;423(6937):255‐260. 12714970 10.1038/nature01572

[5] Dimri GP , Martinez JL , Jacobs JJ , et al. The Bmi‐1 oncogene induces telomerase activity and immortalizes human mammary epithelial cells. Cancer Res. 2002;62(16):4736‐4745. 12183433

[6] van der Lugt NM , Domen J , Linders K , et al. Posterior transformation, neurological abnormalities, and severe hematopoietic defects in mice with a targeted deletion of the bmi‐1 proto‐oncogene. Genes Dev. 1994;8(7):757‐769. 7926765 10.1101/gad.8.7.757

[7] Park IK , Qian D , Kiel M , et al. Bmi‐1 is required for maintenance of adult self‐renewing haematopoietic stem cells. Nature. 2003;423(6937):302‐305. 12714971 10.1038/nature01587

[8] Zhang H , Ding J , Jin J , et al. Defects in mesenchymal stem cell self‐renewal and cell fate determination lead to an osteopenic phenotype in Bmi‐1 null mice. J Bone Miner Res. 2010;25(3):640‐652. 19653817 10.1359/jbmr.090812

[9] Zencak D , Lingbeek M , Kostic C , et al. Bmi1 loss produces an increase in astroglial cells and a decrease in neural stem cell population and proliferation. J Neurosci. 2005;25(24):5774‐5783. 15958744 10.1523/JNEUROSCI.3452-04.2005 PMC6724881

[10] Zhang Q , Li J , Li Y , et al. Bmi deficiency causes oxidative stress and intervertebral disc degeneration which can be alleviated by antioxidant treatment. J Cell Mol Med. 2020;24(16):8950‐8961. 32583517 10.1111/jcmm.15528 PMC7417700

[11] Park IK , Morrison SJ , Clarke MF . Bmi1, stem cells, and senescence regulation. J Clin Invest. 2004;113(2):175‐179. 14722607 10.1172/JCI20800 PMC311443

[12] Meng S , Luo M , Sun HE , et al. Identification and characterization of Bmi‐1‐responding element within the human p16 promoter. J Biol Chem. 2010;285(43):33219‐33229. 20551323 10.1074/jbc.M110.133686 PMC2963359

[13] Molofsky AV , He S , Bydon M , Morrison SJ , Pardal R . Bmi‐1 promotes neural stem cell self‐renewal and neural development but not mouse growth and survival by repressing the p16Ink4a and p19Arf senescence pathways. Genes Dev. 2005;19(12):1432‐1437. 15964994 10.1101/gad.1299505 PMC1151659

[14] Campisi J . Senescent cells, tumor suppression, and organismal aging: good citizens, bad neighbors. Cell. 2005;120(4):513‐522. 15734683 10.1016/j.cell.2005.02.003

[15] Baker DJ , Wijshake T , Tchkonia T , et al. Clearance of p16Ink4a‐positive senescent cells delays ageing‐associated disorders. Nature. 2011;479(7372):232‐236. 22048312 10.1038/nature10600 PMC3468323

[16] Oguro H , Iwama A , Morita Y , Kamijo T , van Lohuizen M , Nakauchi H . Differential impact of Ink4a and Arf on hematopoietic stem cells and their bone marrow microenvironment in Bmi1‐deficient mice. J Exp Med. 2006;203(10):2247‐2253. 16954369 10.1084/jem.20052477 PMC2118102

[17] Bruggeman SW , Valk‐Lingbeek ME , van der Stoop PP , et al. Ink4a and Arf differentially affect cell proliferation and neural stem cell self‐renewal in Bmi1‐deficient mice. Genes Dev. 2005;19(12):1438‐1443. 15964995 10.1101/gad.1299305 PMC1151660

[18] Biehs B , Hu JK , Strauli NB , et al. BMI1 represses Ink4a/Arf and Hox genes to regulate stem cells in the rodent incisor. Nat Cell Biol. 2013;15(7):846‐852. 23728424 10.1038/ncb2766 PMC3735916

[19] Yin Y , Xue X , Wang Q , Chen N , Miao D . Bmi1 plays an important role in dentin and mandible homeostasis by maintaining redox balance. Am J Transl Res. 2016;8(11):4716‐4725. 27904674 PMC5126316

[20] Huang Y , Chen N , Miao D . Pyrroloquinoline quinone plays an important role in rescuing Bmi‐1(^−/−^) mice induced developmental disorders of teeth and mandible anti‐oxidant effect of pyrroloquinoline quinone. Am J Transl Res. 2018;10:40‐53. 29422992 PMC5801345

[21] Jin JL , Tao JG , Gu X , et al. p16Ink4a deletion ameliorated renal tubulointerstitial injury in a stress‐induced premature senescence. Sci Rep. 2017;7:7502. 28790310 10.1038/s41598-017-06868-8 PMC5548892

[22] Molofsky AV , Pardal R , Iwashita T , Park IK , Clarke MF , Morrison SJ . Bmi‐1 dependence distinguishes neural stem cell self‐renewal from progenitor proliferation. Nature. 2003;425(6961):962‐967. 14574365 10.1038/nature02060 PMC2614897

[23] Nakatomi M , Ida‐Yonemochi H , Ohshima H . Lymphoid enhancer‐binding factor 1 expression precedes dentin sialophosphoprotein expression during rat odontoblast differentiation and regeneration. J Endod. 2012;39(5):612‐618. 10.1016/j.joen.2012.12.016 23611378

[24] Yin Y , Wang Q , Sun W , Wang Y , Chen N , Miao D . p27(kip1) deficiency accelerates dentin and alveolar bone formation. Clin Exp Pharmacol Physiol. 2014;41(10):807‐816. 24916068 10.1111/1440-1681.12276

[25] Nicholas WF , Aaron P , David M , Jean G . p53 oligomerization status modulates cell fate decisions between growth, arrest and apoptosis. Cell Cycle. 2016;15(23):3210‐3219. 27754743 10.1080/15384101.2016.1241917 PMC5176156

